# Calcium Pre-Rinse: Effect on permeability of dentin tubules by fluoride rinse

**DOI:** 10.4317/jced.55382

**Published:** 2019-04-01

**Authors:** Tacíria-Machado-Bezerra Braga, David-Nascimento Braga, Edilausson Moreno-Carvalho, José-Roberto-de Oliveira Bauer, Cecilia-Pedroso Turssi

**Affiliations:** 1 DDS, MSc, PhD student, Faculdade São Leopoldo Mandic – Instituto de Pesquisas São Leopoldo Mandic, Campinas, SP, Brazil; Assistant professor, Faculty of Dentistry, CEUMA University, São Luís, MA, Brazil; 2 DDS, Specialist in Oral and Maxillofacial Surgery; 3 DDS, MSc, PhD student, Faculty of Dentistry, Federal University of Maranhão, São Luís, Brazil; 4 DDS, MSc, PhD, Associate Professor, Federal University of Maranhão, São Luís, Brazil; 5 DDS, MSc, PhD, Professor, Faculdade São Leopoldo Mandic – Instituto de Pesquisas São Leopoldo Mandic, Campinas, SP, Brazil

## Abstract

**Background:**

The aim of this study was to evaluate whether calcium (CaL) solution would enhance the capacity of sodium fluoride (NaF) solution in reducing the permeability of hypersensitive dentin.

**Material and Methods:**

Thirty-two Wistar rats ingested for 45 days acidic isotonic drink (Gatorade, pH 2.7) ad libitum to induce dental erosion. Then, molar teeth received a cold stimulus to confirm the presence and score the intensity of dentin hypersensitivity based on body contraction and noise. Animals were allocated to four groups (n=8), according to the solution(s) applied in the oral cavity: NaF (12 mmol/L, 1 min); CaL (150 mmol/L, 1 min); CaL followed by NaF (CaF+NaF, 1 min each); distilled water (DW, 1 min, as negative control). The animals were euthanized and the mandibles dissected into hemimandibles, which were sealed with sticky wax, except for the occlusal surface of the molar teeth. The samples were immersed in 10% copper sulphate solution and in 1% dithioxamide alcoholic solution (25 min each). The samples were sectioned longitudinally and imaged under optical microscope. Then, dentin permeability was measured as the area of copper ion penetration, using ImageJ software. Photomicrographs were obtained by scanning electron microscopy.

**Results:**

68.7% of animals had body contraction associated or not with noise. One-way ANOVA followed by Tukey´s test indicated that groups treated with NaF solution, whether or not preceded by CaL solution, presented lower permeability than the remaining groups [CaL+NaF: 3405.7 μm2 (±1796.4); NaF: 4111.7 μm2 (±2450.6); CaL: 42254.6 μm2 (±30399.2); DW: 37064.6 μm2 (±21994.4)]. Photomicrographs showed that CaL+NaF group presented an increased proportion of occluded dentin tubules in comparison to the NaF-only group.

**Conclusions:**

Although qualitatively there seems to be a benefit in using CaL pre-rinse, this solution did not quantitatively enhance the capacity of NaF in reducing permeability of hypersensitive dentin.

** Key words:**Dentin hypersensitivity, Fluoride, Calcium lactate, Animal model.

## Introduction

Although there has been a decline in the prevalence of caries lesions (1), an increase in non-carious lesions has been observed (2,3). These are mainly the result of erosive, abrasive and fatigue wear (3). Clinically, these lesions may impair functional and aesthetic and cause negative impacts on quality of life (4), since they are associated with dentin hypersensitivity (DH) (5,6).

DH is considered a common oral health problem, affecting about 35% of the population, with higher prevalence among females (7). It is characterized by acute and short duration pain due to the exposure of dentin, usually caused by erosive and/or abrasive lesions, in response to thermal, tactile, evaporative or chemical stimuli (8). In order to eliminate or minimize pain discomfort, there are two treatment approaches: modification or blocking of the pulpal nerve response and occlusion of exposed dental tubules, reducing dentin permeability (8).

Fluoridated products, including sodium fluoride (NaF) solutions, are among the strategies intended to chemically occlude dentin tubules (9). Their mechanism of action relies on the formation of calcium fluoride (CaF2) like minerals, which can partially occlude dentin tubules (10).

Owing to the fact that calcium lactate solution (CaL) pre-rinse enhances NaF effect even under erosive challenges (11,12), it could be assumed that CaL might have the capacity to optimize the protection performed by NaF in managing DH. Thus, the aim of this study was to evaluate whether CaL would enhance the capacity of NaF in reducing the permeability of hypersensitive dentin.

## Material and Methods

-Experimental design

**Figure 1:**
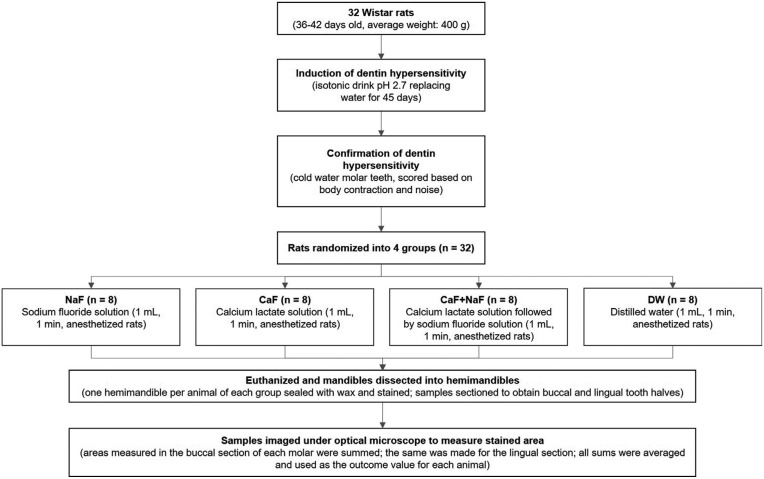
Flowchart of the experiment.

For this study, 32 male Wistar rats (Rattus Norvegicus) with 36-42 days of age and average weight of 400 g (Anilab Laboratory, Paulínia, SP, Brazil) with hypersensitive dentin were allocated to four groups (n = 8) to receive the following solutions: 1) NaF; 2) CaL; 3) CaL followed by NaF (CaL+NaF), and distilled water (DW), as negative control. The response variable was dentin permeability, measured in μm2. Figure 1 depicts the flowchart of the experiment.

-Ethical aspects and conditioning of animals

After approval by the local Ethics Committee (protocol #2016/016), animals were housed in groups of four each in polypropylene cages, bedded with wood shavings, in an open system in ventilated shelves, with controlled temperature of 24°C. Photoperiod was 12 hours light and 12 hours dark, and commercial feed (Nuvital Co., São Paulo, SP, Brazil) was offered ad libitum.

-Induction of dentin hypersensitivity

In order to expose dentin tubules and induce hypersensitivity, the teeth of the animals were eroded using a validated model (13). In brief, animals ingested an acidic isotonic drink (Gatorade, lemon flavor, pH 2.7, Ambev, Jaguariúna, SP, Brazil), replacing drinking water for a period of 45 days (13). Solutions were packed in plastic bottles and changed daily.

On day 46, hypersensitivity induction was confirmed and scored by applying cold stimulus to molar teeth. For this, 0.5 mL of water at 4°C was applied with a syringe equipped with a metal cannula for 5 s on the buccal surface of the left second molar. The animal’s response to pain was classified according to the following scores: 0 = no response; 0.5 = slight body contraction; 1 = body contraction; 2 = strong body contraction and short noise; 3 = strong body contraction and prolonged noise. All 32 animals presented pain signals and were therefore eligible to be randomly allocated to the groups (Fig. 1).

-Treatment application

Animals were allocated to four groups (n = 8), according to the solution(s) applied in the oral cavity: NaF; CaL; CaL+NaF; DW.

Each animal was kept under dissociative anesthesia obtained by intramuscular injection of 50 mg/Kg Zoletil (Zoletil 50, Virbac do Brasil, São Paulo, SP, Brazil). Once anesthetized, each animal was positioned in dorsal decubitus, with the front and hind paws maintained in abduction, using a metal device. Elastics were gently attached to the upper and lower incisors, which kept the oral cavity open for treatment.

The oral cavity of the animals pertaining to the NaF group was exposed to 1 mL of a 228 ppm (12 mmol/L) NaF solution for 1 min, while in the CaL group, a 150 mmol/L CaL solution was applied in the same volume and for the same 1-min duration. In the group in which NaF was preceded by CaL (Cal+NaF), both solutions were applied as previously described. The oral cavity of the rats of the negative control group was exposed to distilled water. Solutions or distilled water was sucked from the oral cavity using 5-mL syringe and the teeth were gently dried with a gauze. In all groups, exposure to the respective treatment was performed in a single occasion.

-Euthanasia of animals 

Immediately after treatment, each animal was euthanized with intramuscular injection of ketamine solution (200 mg/Kg body weight) and intraperitoneal injection of thiopental and lidocaine in the proportion of 150 mg/Kg to 1 mg/mL, respectively (14). The mandible were dissected, soft tissue trimmed and sectioned at the midline into hemimandibles.

-Dentin permeability

The effect of the treatments on dentin permeability was analyzed using a histochemical staining method (15). One hemimandible per animal of each group was sealed with stick wax in order to keep exposed only the occlusal surface. Subsequently, the hemimandibles were individually immersed in 10 mL of 10% copper sulphate solution for 25 min in an oven at 37±0.5ºC, dried on absorbent paper and immersed in 1% dithioxamide alcoholic solution for 25 min. Finally, hemimandibles were rinsed with distilled water for 10 s, dried and individually kept in a sealed container with cotton soaked in 0.5 mL ammonia for 7 days. Each stained hemimandible was fixed on an acrylic plate and sectioned longitudinally through its center to obtain sections of the buccal and lingual molars’ halves using a precision saw (Isomet 1000, Buehler, Lake Bluff, IL, USA).

The cut surface of the molars of the two sections (buccal and lingual) of each hemimandible were imaged using an optical microscope (Nikon Eclipse-Ci Infinity 1-3C, Tokyo, Japan) under 40x magnification. The area presenting copper ions revealed by dithioxamide was measured in pixels and converted into µm2 using ImageJ (National Institute of Health, Bethesda, MD) by a single previously trained examiner. Each molar of the buccal section had their measured areas summed. The same was made for the lingual section. Then, all sums were averaged and used as the outcome value for each animal. The lower the penetration area, the lower the dentin permeability.

-Photomicrographs in scanning electron microscopy (SEM)

Sections representative of each group were allowed to dry in a desiccator for 48 h, fixed in sample holder and analyzed under 50x, 250x, 500x, 1,000x, 2,000x and 5,000x magnifications using a scanning electron microscope (TM3030, Hitachi Chiyoda, Tokyo, Japan) operating at 15 KV. SEM photomicrographs were qualitatively evaluated for the presence, proportion of occluded dentin tubules and amount of deposits on dentin surface and tubules.

-Statistical analysis

The scores of DH-induced pain data were described by means of absolute and relative frequencies and median. The effect of treatments tested was evaluated using one-way analysis of variance and Tukey’s test. The level of significance was set at 5%. Statistical calculations were carried out with SPSS 23 (SPSS Inc., Chicago, IL, USA).

## Results

**Figure 2:**
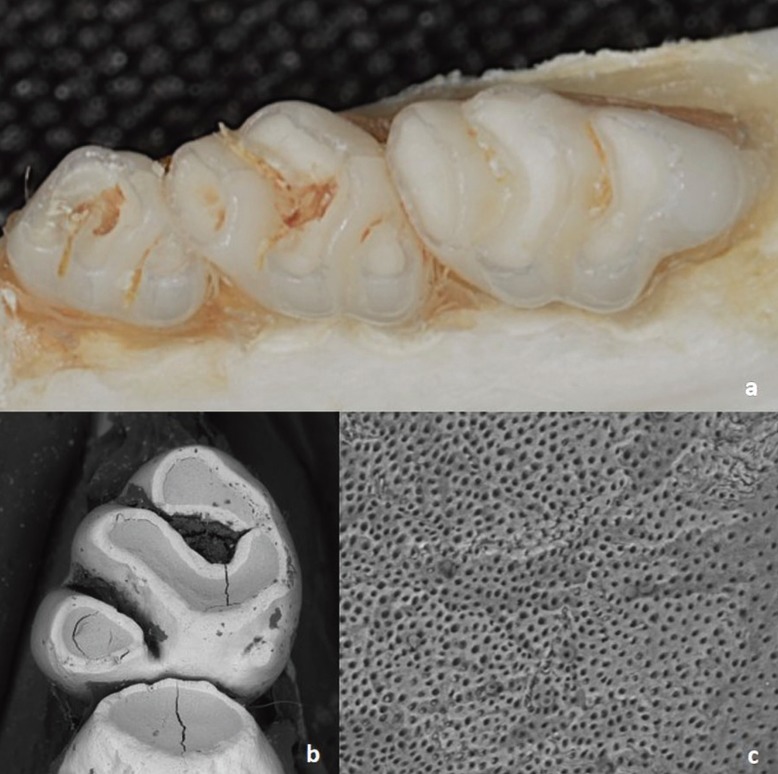
Worn occlusal surface after erosion caused by the acidic isotonic drink (a), showing dentin exposure (b) an patent tubules (c).

While confirming DH induction, it was noticed that 68.7% of the rats had body contraction associated or not with noise (scores 1 to 3). Considering all animals, the pain median was 2 ( Table 1). Figures 2a through c depict the exposure and wear of dentin surface and patent tubules 45 days after the consumption of the acidic isotonic drink.

**Table 1:**
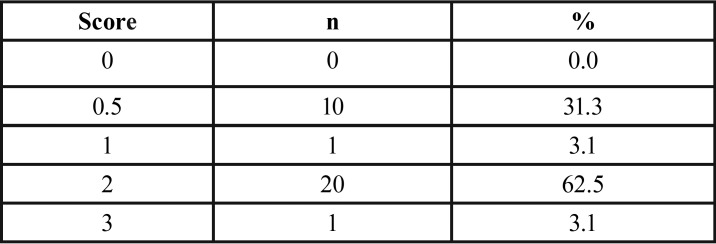
Absolute (n) and relative (%) frequency of pain scores after the induction of dentin hypersensitivity.

One-way analysis of variance demonstrated statistically significant difference between groups regarding dentin permeability (*p* < 0.001, with 99.4% statistical power). The Tukey’s test showed that dentin permeability was lower in the NaF-treated groups, whether or not preceded by CaL, in comparison to the CaL and negative control (DW) group ( Table 2).

**Table 2:**
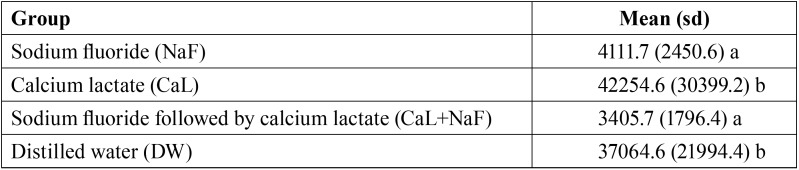
Dentin permeability of each group presented as the mean (standard deviation) of the measurement of the stained area (µm2).

**Figure 3:**
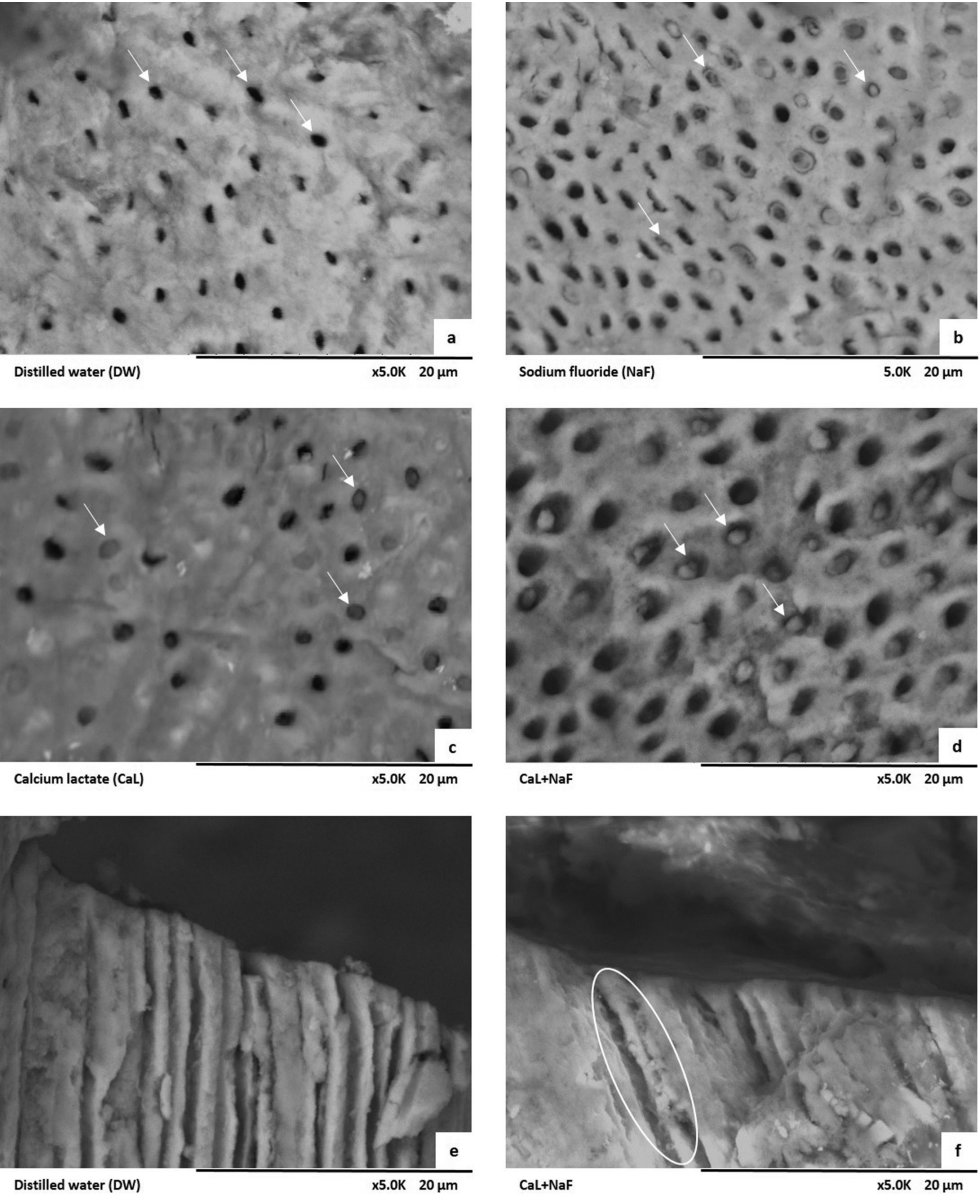
Scanning electron microscopy images (5,000x and 2,000x magnification) showing the negative control group (a), with patent tubules (arrows); NaF, CaL and CaL+NaF groups with partially occluded dentin tubules (b, c, d); inside dentin tubules of the negative control group (e) with no deposits; and CaL+NaF group (f) with deposits partially occluding tubules.

Photomicrographs showed that the negative control group (DW) had patent tubules (Fig. 3a). In the NaF-treated group, there was partial occlusion of dentin tubules (Fig. 3b), while in CaL and CaL+NaF groups a higher proportion of tubules were occluded (Fig. 3c,d). In contrast to the negative control group (Fig. 3e), CaL+NaF group showed deposits inside the dentin tubules (Fig. 3f).

## Discussion

NaF solution has been part of the strategies to manage dentin hypersensitivity through the formation of deposits of CaF2 globules, which can physically occlude dentin tubules (10). Although dentin has the advantage to show three times higher the concentration of CaF2 deposits than enamel, they are unstable and desadsorb (16). In order to look towards the possibility of potentiating the effect of NaF solution in the context of DH, in this study we investigated whether CaL pre-rinse would reduce the permeability of hypersensitive dentin.

As the creation of erosive lesions under controlled conditions to induce DH would be unfeasible in humans,an animal model was adopted. Rats were used because the number of dentin tubules found in rat dentin is comparable to those found in human dentin (17). By using a rat model previously described (13), DH was developed after the creation of erosive lesions using an acidic isotonic drink.

It is worth mentioning that the erosive potential of the isotonic drink used in this study has also been demonstrated in laboratory studies (18,19). In addition to the low pH and high titratable acidity (20), the erosive potential of the isotonic drink has been attributed to its reduced Ca content (19). Figures 2a and b substantiates the surface loss of the rats’ molars and dentin exposure caused by the intake of acidic isotonic drink.

The induction of DH was confirmed by the animal’s response to pain to cold water application on their molar teeth, corroborating the results found elsewhere (13). In the present research, most of the animals, as in the quoted study, presented the second highest pain score (score 2) in the sensitivity scale. Thus, the results found confirmed that the isotonic drink intake, in fact, induced DH.

The exposed dentin were then treated with NaF, CaL, CaL+NaF or DW in order to compare their capacity to increase tubule occlusion, i.e, reduce dentin permeability. It was observed that the lowest permeability values was seen after NaF treatment, whether or not preceded by CaL solution. In fact, as can be seen in the SEM photomicrographs, dentin tubules was partially occluded in both NaF-treated groups. This result is similar to *in vitro* and *in vivo* studies that evaluated the efficacy of fluoride products in the management of DH and found that these products decrease dentin permeability but produced only partial occlusion of dentin tubules (17,21).

The fact that CaL did not enhance the effect of NaF in reducing dentin permeability can be explained by the discrepancy between the dentine tubule size and CaF2 globules, which are smaller. Thus, one can hypothesize that even in the presence of deposits of CaF2 globules on intertubular dentin and inside the dentin tubules, diffusion of cooper sulphate occurred through spaces between globules. Although one cannot rule out that the single application of the CaL pre-rinse may have contributed to the lack of its benefit in enhancing the NaF action, the single application was intended to avoid the necessity of multiple anesthesia to apply the solutions in the oral cavity of the animals. Further studies are warranted to evaluate whether the application of CaL+NaF repeatedly would promote an improvement in the occluding capacity of dentin tubules, reducing dentin permeability.

By examining the SEM photomicrographs one can notice that samples treated with DW water showed patent dentin tubules. In contrast, in the NaF-treated groups, associated or not to CaL, there was partial occlusion of various dentin tubules. It can also be seen that the CaL+NaF group presented an increased proportion of occluded dentin tubules in comparison to the NaF-only group. When evaluating inside the tubules of the CaL+NaF group, deposits of mineral content was observed, causing partial obliteration, unlike the DW group, in which tubules are patent.

Several studies have evaluated the effect of Ca pre-rinse associated with NaF on caries control (22,23) and against erosive episodes (11,12,24) with promising results. According to the current findings, although CaL solution seems to increase tubular occlusion provided by the NaF solution, its effectiveness in controlling permeability of hypersensitive dentin was not statistically superior to that found for the NaF-only group. One should bear in mind, however, that although CaF solution did not significantly enhance the action of NaF solution in reducing dentin permeability, CaL+NaF made dentin approximately 21% less permeable than the NaF-only. Yet the impact of such reduction remains unknown, chances are that clinically it may be related to a decrease in hypersensitivity intensity.

According to the adopted animal model, it was concluded that although qualitatively there seems to be a benefit in using CaL pre-rinse, this solution did not quantitatively enhance the capacity of NaF in reducing permeability of hypersensitive dentin.
